# LSD1 dual function in mediating epigenetic corruption of the vitamin D signaling in prostate cancer

**DOI:** 10.1186/s13148-017-0382-y

**Published:** 2017-08-11

**Authors:** Sebastiano Battaglia, Ellen Karasik, Bryan Gillard, Jennifer Williams, Trisha Winchester, Michael T. Moser, Dominic J Smiraglia, Barbara A. Foster

**Affiliations:** 1 Center for Immunotherapy, Roswell Park Cancer Institute, Elm and Carlton St, Buffalo, NY 14263 USA; 2 Department of Pharmacology and Therapeutics, Roswell Park Cancer Institute, Elm and Carlton St, Buffalo, NY 14263 USA; 3 Department of Cancer Genetics, Roswell Park Cancer Institute, Elm and Carlton St, Buffalo, NY 14263 USA

## Abstract

**Background:**

Lysine-specific demethylase 1A (LSD1) is a key regulator of the androgen (AR) and estrogen receptors (ER), and LSD1 levels correlate with tumor aggressiveness. Here, we demonstrate that LSD1 regulates vitamin D receptor (VDR) activity and is a mediator of 1,25(OH)_2_-D_3_ (vitamin D) action in prostate cancer (PCa).

**Methods:**

Athymic nude mice were xenografted with CWR22 cells and monitored weekly after testosterone pellet removal. Expression of LSD1 and VDR (IHC) were correlated with tumor growth using log-rank test. TRAMP tumors and prostates from wild-type (WT) mice were used to evaluate VDR and LSD1 expression via IHC and western blotting. The presence of VDR and LSD1 in the same transcriptional complex was evaluated via immunoprecipitation (IP) using nuclear cell lysate. The effect of LSD1 and 1,25(OH)_2_-D_3_ on cell viability was evaluated in C4-2 and BC1A cells via trypan blue exclusion. The role of LSD1 in VDR-mediated gene transcription was evaluated for *Cdkn1a*, *E2f1*, *Cyp24a1*, and *S100g* via qRT-PCR-TaqMan and via chromatin immunoprecipitation assay. Methylation of Cdkn1a TSS was measured via bisulfite sequencing, and methylation of a panel of cancer-related genes was quantified using methyl arrays. The Cancer Genome Atlas data were retrieved to identify genes whose status correlates with LSD1 and DNA methyltransferase 1 (DNMT1). Results were correlated with patients’ survival data from two separate cohorts of primary and metastatic PCa.

**Results:**

LSD1 and VDR protein levels are elevated in PCa tumors and correlate with faster tumor growth in xenograft mouse models. Knockdown of LSD1 reduces PCa cell viability, and gene expression data suggest a dual coregulatory role of LSD1 for VDR, acting as a coactivator and corepressor in a locus-specific manner. LSD1 modulates VDR-dependent transcription by mediating the recruitment of VDR and DNMT1 at the TSS of VDR-targeted genes and modulates the epigenetic status of transcribed genes by altering H3K4me2 and H3K9Ac and DNA methylation. Lastly, LSD1 and DNMT1 belong to a genome-wide signature whose expression correlates with shorter progression-free survival and overall survival in primary and metastatic patients’ samples, respectively.

**Conclusions:**

Results demonstrate that LSD1 has a dual coregulatory role as corepressor and coactivator for VDR and defines a genomic signature whose targeting might have clinical relevance for PCa patients.

**Electronic supplementary material:**

The online version of this article (doi:10.1186/s13148-017-0382-y) contains supplementary material, which is available to authorized users.

## Background

Prostate cancer (PCa) is the most commonly diagnosed cancer in men in the USA and the second leading cause of cancer death [1]. The secosteroid hormone 1,25-dihydroxy-vitamin D_3_ (1,25-D_3_) binds to the vitamin D receptor (VDR), which translocates into the nucleus, binds to VDR-responsive elements (VDREs), and associates with coregulatory complexes to either activate or repress gene transcription. 1,25-D_3_ is metabolized by the VDR target gene CYP24A1 [2] and activates a number of downstream metabolic pathways including calcium absorption through induction of S100g [3] and maintenance of bone health [4–6] and cellular pathways regulating cell differentiation and proliferation [7–10]. 1,25-D_3_-bound VDR can cause cell cycle arrest by targeting G0S2, CDKN1A [11, 12], IGFBP3 [13], and E2F target genes [14]. Furthermore, 1,25-D_3_ can induce apoptosis by repressing WNT/β-catenin signaling, as shown in Vdr^−/−^ mice [15], and by targeting c-Myc [16] and inhibiting the expression of the anti-apoptotic genes Bcl2 and BclXL [17]. As 1,25-D_3_ has a potent antiproliferative effect on prostate epithelial cells, late-stage PCa frequently loses sensitivity to numerous nuclear receptor (NR) ligands, including 1,25-D_3_ [18, 19]. Numerous mechanisms describing loss of 1,25-D_3_ responsiveness have been proposed including VDR mutations [20–22], differential recruitment of coregulatory proteins [19, 23–25], and changes in the epigenetic landscape of tumor cells [26–29].

The lysine-specific demethylase 1A (LSD1/KDM1A) is a demethylating enzyme that targets mono- and dimethyl-H3K4 [30], associated with open chromatin structure permissive to transcription, and dimethyl-H3K9 [31], associated with close chromatin structure and transcriptional repression. LSD1 also targets non-histone proteins such as TP53, repressing p53-targeted gene activation [32], and DNA methyltransferase 1 (DNMT1), promoting protein stability and maintenance of CpG methylation [33]. LSD1 is overexpressed in numerous cancers including bladder [34], breast [35], brain [36], and prostate [31], underlying LSD1 clinical relevance across multiple malignancies. Given the frequency of overexpression as well as its role in regulating transcriptional outcomes of NRs, LSD1 is being investigated as a novel clinical target for cancer patients with promising in vitro results [36, 37].

In this study, we describe for the first time LSD1 acting as transcriptional corepressor and coactivator for VDR, and we propose a mechanism by which LSD1 modulates VDR-dependent epigenetic regulation of gene transcription. We also define DNMT1 as a key player in the epigenetic changes regulated by LSD1, ultimately defining a VDR/LSD1/DNMT1 genomic signature that correlates with clinical outcome of patients with PCa.

## Methods

### Cell culture and treatments

BC1A cells used in this manuscript were isolated by BF and MM from a bone metastasis in a TRAMP mouse (FVB:C57BL/6 50:50 strain background) [38, 39]. BC1A cells were established by three rounds of clonal dilutions and were used as a model for 1,25-D_3_ resistance. C4-2 cells were obtained from Dr. Leland Cheung (Cedars-Sinai Medical Center), and CWR22 cells were a kind gift from Dr. Thomas G. Pretlow (Case Western Reserve University). C4-2 and CWR22 cells were validated via microsatellite PCR at the Roswell Park Cancer Institute (RPCI) Genomics Core. BC1A cells are a murine in-house-derived cell line. BC1A cells were maintained in DMEM (Gibco) media supplemented with 10% FBS (Gibco, 26,140–079), 5 μg/mL of insulin (Invitrogen, 12585-014), 10 nM DHT (Sigma, D-073-1ML), and penicillin/streptomycin (Invitrogen, 15140-122). C4-2 cells were maintained in RPMI1640 (Gibco) with 10% FBS and penicillin/streptomycin. CWR22 were used only for in vivo experiments, as described below.

### Mice and tumor xenograft

This study was approved by the RPCI and Institutional Animal Care and Use Committee (RPCI/IACUC) and carried out by the Mouse Tumor Model Resource (MTMR) Core at RPCI. Six-week-old athymic nude (Hsd:Athymic Nude-Foxn1nu) mice (*n* = 50) were purchased from Envigo (069(nu)/070(nu/+)). The mice were castrated, and testosterone (Sigma T1500-25G) pellets (13 mg/mouse) were implanted subcutaneously. The mice were subsequently injected subcutaneously with 10^6^ CWR22 cells in matrigel (Corning 354234). Tumors were assessed weekly via caliper measurement, and tumor volume was calculated using the following formula: *[(dim*_*(short)*_ *)* ^*2*^ ]*[dim_*(long)*_ ]*0.5324. Testosterone pellets were removed (castration) once tumors reached 200 mm^3^, and tumor volume was measured weekly for 45 weeks. Mice that never developed tumors or mice that died before pellet removal were excluded from the study. The mice were euthanized either when signs of toxicity appeared (lethargy, paleness, hunched back, loss of weight) or when tumors reached 2 cm in any direction as per institutional guidelines. At the time of death, tumors were resected and utilized for immunohistochemistry analysis.

### Immunohistochemistry (IHC)

Tissues were fixed in 10% buffered formalin for 24 h prior to processing. Tissues were processed and embedded in paraffin and then sectioned at 5 μm. Slides were deparaffinized in several baths of xylene and then rehydrated in graded alcohols followed by ddH_2_O. Slides were incubated in 1× pH 6 citrate buffer (Invitrogen, 00-5000) in DAKO PT Link for 20 min. IHC was performed using DAKO Autostainer Plus following manufacturer’s instructions. Slides were incubated in 3% H_2_O_2_ for 15 min. To block non-specific binding, tissues were incubated with 10% normal goat serum for 30 min, followed by avidin/biotin block (Vector Labs, SP-2001). Primary antibody LSD1 (Cell Signaling, 2139) or VDR (Thermo Scientific MA1710 (Clone 9A7)) were diluted in 1% BSA solution and incubated for 30 min at room temperature, followed by the biotinylated Goat Anti-Rabbit secondary antibody (Abcam, ab6720) for 15 min. For signal enhancement, ABC reagent (Vector Labs, PK-6100) was applied for 30 min. Slides were then incubated with DAB substrate (Dako, K3467) for 5 min and then counterstained with DAKO hematoxylin for 20 s. Slides were dehydrated through several baths of graded alcohols and xylenes and then coverslipped. Finally, slides were scanned using the Aperio System (Leica).

### Western blotting

When murine tissues were used, the dorsal, lateral, and ventral prostates were microdissected from wild-type and TRAMP mice. The prostates were resuspended in 300 μl RIPA buffer and minced using tissue homogenizer (Polytron, PT1035). Lysates were centrifuged at 16,000 rpm for 10 min at + 4 °C, and clear supernatant was recovered. When cell lines were used, nuclear and cytoplasmic lysates were extracted using the NE-PER kit (Thermo Scientific, 78833) following the manufacturer’s instructions. Protein concentration was quantified using the Bio-Rad RC-DC protein assay kit (Biorad, 500-0121). Forty micrograms of protein were loaded onto a 10% SDS gel (Biorad) and ran for 90 min at 120 V. Proteins were transferred onto a PVDF membrane (Invitrogen, LC2002) using Biorad transfer buffer (Biorad 161-0771) for 110 min at 80 V at + 4 °C. Next, the membrane was blocked with 5% milk in TBST for 1 h at RT, incubated overnight with LSD1 (Cell Signaling 2139), VDR (Thermo Scientific MA1710 (Clone 9A7)), or GAPDH (Cell Signaling 2118) primary antibody, incubated with secondary HRP-conjugated secondary antibody (Santa Cruz sc-2030) and developed using chemoluminescent reaction (Pierce, 32132).

### Immunoprecipitation (IP)

Nuclear proteins were extracted using the NE-PER Nuclear and Cytoplasmic Extraction Kit (Thermo Scientific, 78833), and lysates were pre-cleared with A/G agarose beads (Millipore, LSKMAGA02) for 1 h at 4 °C. LSD1 antibody (Cell Signaling 2139) at 1:50 dilution and IgG Rabbit (Santa Cruz, 2027) at 1:500 dilution were added to each sample and incubated on a rotator over night at 4 °C. The pulldown of LSD1 antibody and IgG control was achieved by adding A/G agarose beads to the samples and incubating them on a rotator for 3 h at 4 °C. Samples were then used for western blot analysis using the following antibodies: LSD1 (Cell Signaling, 2139) and VDR (Santa Cruz Sc-13133 (Clone D-6)).

### Survival analysis

Survival analysis for mice with high/low LSD1 and VDR tumor protein levels was done using *R* and the package survival. IHC slides were scored as “low” (negative/low staining) or positive (medium/strong staining), and the mice were grouped accordingly. Since tumor reaching 1000 mm^3^ tends to keep proliferating till reaching the size limit imposed by the RPCI LAR/IACUC (2 cm in any dimension), tumor recurrence was defined as tumor reaching 1000 mm^3^ after castration. Data were analyzed in *R* [40] with the survival package [41], and log-rank test was used to assess the difference in time to recurrence in the high vs. low groups with a significance threshold of *p* value < 0.05.

### Cell transfection

*siRNA transfection*: BC1A cells were transfected with small interfering RNA (siRNA) for LSD1 or scrambled siRNA control (Ambion, s97504 and 12935-200) using Lipofectamine 2000 (Invitrogen, 11668027), following the manufacturer’s instructions. Briefly, siRNA and Lipofectamine were diluted in OptiMEM (Invitrogen 11058021); siRNA/lipid complexes were then added to BC1A cells in the OptiMEM with 5% FBS. Cells were incubated overnight before replacing media with full DMEM. *shRNA transfection*: BC1A and C4-2 cells were prepared at the RPCI small hairpin RNA (shRNA) Core facility. Briefly, the cells were stably transfected with scrambled shCTR (Dharmacon, RHS4346) and murine shLSD1 (Dharmacon, RMM4431) for BC1A or human shLSD1 (Dharmacon, RHS4430) for C4-2 cells. Cells transfected with shRNA were maintained in complete media with 2 μg/mL of puromycin (Invitrogen, A1113803).

### Cell viability assay

BC1A cells transfected with siCTR/siLSD1 or C4-2 cells transfected with shCTR/shLSD1 were treated with 100 nM 1,25-D_3_ or vehicle control and incubated for 72 h at 37 °C. Cells were collected using trypsin (Sigma, T4049), and viable cells were counted via trypan blue exclusion using the automatic cell counter (Beckman Coulter, ViCellXR). Cell counts were normalized to vehicle-treated cells, and statistical significance was calculated using two-way ANOVA with post hoc Tukey test using GraphPad Prism.

### Gene expression analysis

BC1A cells transfected with siCTR/siLSD1 were treated for 4 or 24 h with 100 nM 1,25-D_3_. Media was removed, and the cells were washed and resuspended in 1 mL of TRI Reagent (Ambion, AM9738). RNA was extracted following the manufacturer’s directions. RNA was quantified using ThermoScientific NanoDrop 8000, and 1 μg of RNA was reverse-transcribed into cDNA using the SuperScript First Strand Synthesis kit (Invitrogen, 11904-018). qRT-PCR-TaqMan primers for Lsd1, E2f1, Cdkn1a, Cyp24a1, and S100g were ordered from Applied Biosystems (Mm01181042_m1 Mm00432936_m1, Mm00432448_m1, Mm00407244_m1, and Mm00486654_m1, respectively). qRT-PCR universal MasterMix (Roche) was used. Data were normalized to beta-actin (Applied Biosystems, Mm00607939_s1) and fold changes (FC) calculated using the 2^-ddCt formula using shCTR vehicle treated as control. Statistical significance was evaluated by two-way ANOVA with post hoc Tukey test using the GraphPad Prism. For tissues, mRNA from the ventral, lateral, and dorsal prostates was extracted by homogenizing the tissues and using TRI Reagent, following the manufacturer’s instructions. Five samples per age group were used.

### Chromatin immunoprecipitation

Primers are listed in Additional file 1: Table S3. Exponentially growing shLSD1-BC1A and shCTR-BC1A cells were incubated with 100 nM 1,25-D_3_ for 24 h, crosslinked with 1% formaldehyde, and incubated with l-glycine to stop the reaction. Cell pellet was collected in cold PBS and nucleic extract prepared using the MC lysis buffer (10 mM TrisHCl pH 7.5, 10 mM NaCl, 3 mM MgCl_2_, 0.5% (*v*/*v*) NP-40). After centrifugation, the pellets were resuspended in MNase digestion buffer (10 mM TrisHCl pH 7.5, 10 mM NaCl, 3 mM MgCl_2_, 1 mM CaCl_2_, 4% (*v*/*v*) NP-40, 1 mM phenylmethane sulfonyl fluoride (PMSF)) and incubated with 100 units of micrococcal nuclease enzyme for 11 min at 37 °C. Per each sample, digestion was stopped with the addition of EGTA, PMSF, protease inhibitor, SDS, and NaCl. Following 5 min sonication (Bioruptor), the samples were centrifuged at a maximum speed for 10 min at + 4 °C and the supernatant was collected for further analysis. Two hundred microliters of the supernatant were diluted with 300 μL dilution buffer (0.01% SDS, 1.1% Triton X-100, 1.2 mM EDTA, 16.6 mM TrisHCl pH 8.1, 167 mM NaCl) and incubated with the primary antibody overnight at + 4 °C on a rotating platform. The antibodies used are the following: IgG (Santa Cruz, sc-2027/sc2025), histone 3 lysine 4 dimethylated (H3K4me2—Abcam, ab32356), histone 3 lysine 4 acetylated (H3K9Ac—Abcam, ab4441), DNA methyltransferase 1 (DNMT1—Abcam, ab92453), and vitamin D receptor (VDR—Santa Cruz, sc1008-x). Antibody-protein complexes were retrieved using MagnaCHIP magnetic beads (Millipore, 16-663) after washes of 5 min each with high salt buffer, low salt buffer, LiCl buffer, TE buffer, and elution buffer (0.1 M NaHCO_3_, 1% SDS). Finally, DNA was extracted using standard phenol chloroform protocol. Quantitation of the DNA fragments was done using qRT-PCR-SYBRGREEN with the Universal SYBR Green Mastermix (Bio-Rad, 172-5124). Ct values were normalized to the INPUT control and plotted as percentage of INPUT. Statistical significance was evaluated by two-way ANOVA with post hoc Tukey test using the GraphPad Prism.

### DNA methylation analysis

Stably transfected BC1A cells were plated in a 6-well plate and treated for 24 h with 100 nM 1,25-D_3_ or vehicle control. DNA was extracted using the DNeasy kit from Qiagen following the manufacturer’s directions. DNA was bisulfite-treated using the EZ DNA Methylation kit (Zymo Research, D5002), amplified (p21FW: aggaagagagGGTGAAGGAGTGGGTTGGTTT, p21RW: cagtaatacgactcactatagggagaaggctTCCACTCATCACCACACACA), cloned using the TA cloning kit (Invitrogen), and sequenced at the RPCI Sequencing Core. DNA for the methylation array was digested following SABiosciences instructions to create samples corresponding to undigested, methylation-sensitive and methylation-dependent, and both conditions. Qiagen arrays were run on an ABI 7900 thermo cycler following the manufacturer’s guidelines. Data analysis was carried out using the template analysis file offered by SABiosciences. The analysis includes quality control steps that evaluate the quality of the enzymatic digestion and reports the percentage of methylation per each gene. Differential methylation analysis was done by building linear models comparing the average methylation levels in two conditions using *R* and calculating the 95th percentile intervals. Genes whose average methylation levels fell outside the 95th percentiles were deemed significant.

### Regulome Explorer analysis, TCGA data retrieval, and functional enrichment analysis

The Regulome Explorer tool is freely available at http://explorer.cancerregulome.org/. It contains gene expression, DNA methylation, CNVs, SNPs, miRNA expression, and clinical data for the samples available through TCGA (http://cancergenome.nih.gov/) on July 2016. The analysis was performed with 333 PCa clinical samples. Using a gene name (or more), the tool retrieves all the data that significantly correlate with the data available for the query gene(s). Statistical significance of each pairwise association (i.e., LSD1 vs. all and DNMT1 vs. all) is assessed using rank-ordered data and a statistical test appropriate to each data type pair, e.g., Fisher’s test (categorical-categorical), F statistic (continuous-continuous), and ANOVA (continuous-categorical). The top 20 significantly correlated genes were used to query the freely accessible cBioPortal tool to query the TCGA provisional (491 samples under “All Complete Tumors”) and Metastatic Michigan (61 samples under “tumors with sequencing and CNA”) data (http://www.cbioportal.org/index.do). Functional enrichment analysis was run with the Broad’s molecular signature database (http://software.broadinstitute.org/gsea/index.jsp) with a false discovery rate (FDR) < 0.05.

## Results

### LSD1 and VDR are upregulated in advanced and CR-PCa samples

**Fig. 1:**
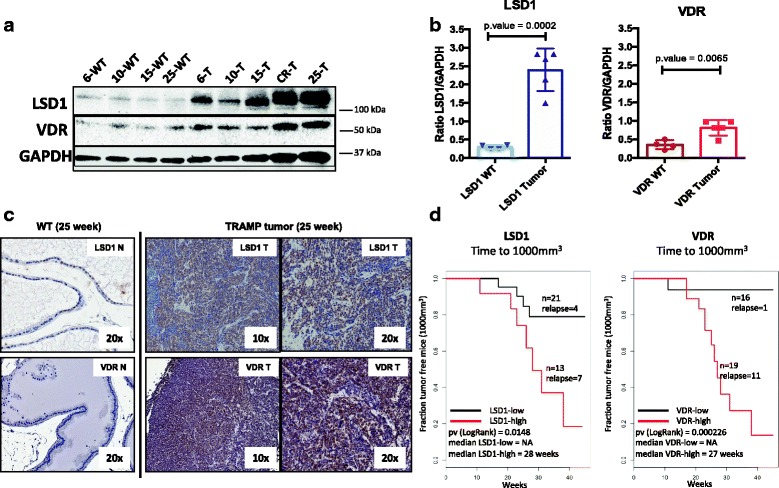
LSD1 expression in prostate tissues is increased in advanced prostate tumors. Western blot and immunohistochemistry (IHC) staining were used to measure protein levels of LSD1 and VDR in wild-type (WT) and TRAMP mice. CWR22 xenograft mice were used to investigate the role of LSD1 and VDR in PCa growth kinetics. **a** Western blotting image showing the expression of LSD1 and VDR protein levels in wild-type and TRAMP prostate lysates. *WT* wild-type mouse, *T* tumor/TRAMP mouse, *CR* castration-recurrent tumor from TRAMP mouse. **b** LSD1 (*left*) and VDR (*right*) protein quantification of LSD1, or VDR, normalized to GAPDH. Data from wild-type samples were compared with the data from tumor samples using Student’s *t* test. *p* values are indicated in the plot. **c** LSD1 and VDR IHC staining in age-matched prostate samples of 25-week-old TRAMP and WT mice. Staining shows a strong nuclear localization, in *brown*, in both WT and TRAMP tumors, with a stronger signal in tumor. *Labels* in the image indicate protein (LSD1, VDR), magnification (× 10, × 20), and tissue type (WT, tumor (T)). **d** Kaplan-Meier plots showing time to recurrence for CWR22 xenografts, measured as time necessary for the tumor to reach 1000 mm^3^ in volume. The *X*-axis indicates weeks of the experiment where time 0 is the time of testosterone pellet removal. The *Y*-axis indicates the percentage of mice with tumor that did not reach 1000 mm^3^. The *black lines* indicate mice with low LSD1/VDR levels, and the *red* lines indicate mice with high LSD1/VDR levels measured via IHC. Log-rank *p* value and median time to recurrence are indicated in the figure

LSD1 protein levels were evaluated in age-matched wild-type (WT) and TRAMP mice by immunohistochemical (IHC) and western blot (WB) analysis. LSD1 mRNA levels moderately vary during disease progression with less than a twofold change ever detected (Additional file 1: Figure S1). We then used whole cell lysates from WT and TRAMP prostates to quantify LSD1 and VDR protein expression. While LSD1 and VDR abundance does not vary in WT samples (Fig. 1a), TRAMP prostates show elevated expression of both proteins, with the highest levels observed in late-stage disease between 15 and 25 weeks of age and in castration-recurrent (CR) tumors (Fig. 1a). Overall, the expression pattern of LSD1 and VDR, normalized to GAPDH, was higher in TRAMP tumors than in WT prostates (Fig. 1b) (LSD1 *p* = 0.0002, VDR *p* = 0.0065). Interestingly, two isoforms of LSD1 were detected in the tumor samples, in accordance with the previously published data [42]. We then evaluated via IHC the cellular localization, which showed a strong nuclear staining for LSD1 and VDR in poorly differentiated PCa (Fig. 1c), corroborating the WB result. The findings in our system are in accordance with the previously published data [43–45], suggesting that LSD1 and VDR levels increase during disease progression in vivo.

### LSD1 and VDR levels correlate with tumor recurrence in vivo

To evaluate whether increased expression of LSD1 and VDR affects the rate of tumor growth and tumor recurrence in vivo, CWR22 cells were injected subcutaneously in castrated athymic nude mice implanted with testosterone pellets. Once the tumors reached 200 mm^3^, androgen deprivation therapy (ADT) was mimicked by surgically removing the testosterone pellets and the tumor volume was monitored weekly. At the end of the study, resected tumors were stained with VDR or LSD1 antibodies and mice were scored as “high” or “low” based on the intensity of the staining (Additional file 1: Figure S2). Censored survival analysis was performed to evaluate whether tumors in mice with high levels of LSD1 or VDR grew faster than those in mice with low levels of LSD1 or VDR, measured as time for the tumor to reach 1000 m^3^ from the time of pellet removal. Results show that mice with high levels of either LSD1 or VDR have significantly shorter time to event than mice with low levels of LSD1 or VDR (log-rank *p* = 0.0148 (LSD1), *p* = 0.00026 (VDR)) (Fig. 1d). These results suggest that LSD1 and VDR might contribute to the development of CR-PCa in vivo by promoting the establishment of a proliferative phenotype.

### LSD1 and VDR belong to the same transcriptional complex

**Fig. 2:**
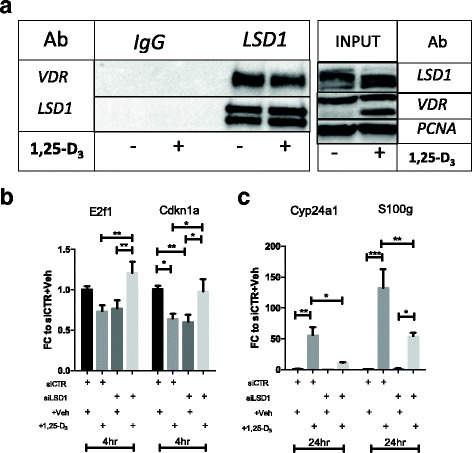
**a** Immunoprecipitation (IP) and western blotting (WB) data showing that LSD1 and VDR belong to the same transcriptional complex. IP was performed from nuclear lysate in samples treated with vehicle control or 1,25-D_3_ using the same LSD1 antibody described for IHC and WB. In post-IP, the samples were probed for LSD1 and VDR. The same double band visible in Fig. 1 was also detected in this sample. **b**, **c** Effect of LSD1 knockdown and 1,25-D_3_ treatment on gene expression of VDR target genes. Every graph compares the effect of vitamin D in control (CTR) cells and LSD1 knockdown (siLSD1) cells. Each *bar* is the mean of at least three biological replicates with SEM, showing the fold changes of treated (+ D3) vs. vehicle-treated (− Veh) samples. The *columns* indicate, from *left* to *right*, siCTR + Veh, siCTR + 1,25-D_3_, siLSD1 + Veh, and siLSD1 + 1,25-D_3_. Transcript levels were measured for **b** *E2f1* and *Cdkn1a* and **c** *Cyp24a1* and *S100g.* Statistical significance was evaluated with one-way ANOVA and Tukey post hoc correction (***p < 0.001, **p < 0.01, *p < 0.05)

In order to assess whether LSD1 and VDR belong to the same macro-molecular complex, LSD1 was pulled down in BC1A cells after 24-h exposure to 100 nM 1,25-D_3_ or vehicle control and samples were probed for VDR. BC1A is a clonal cell line isolated from a bone metastasis in a TRAMP mouse in a FVB:C57BL/6 50:50 strain background and was chosen because it was derived from a naturally developed, and rare, bone metastasis in an in vivo model of PCa. To avoid introducing technical biases, we utilized the same LSD1 antibody used for western blotting and IHC analysis. As in the previous blotting experiment, two isoforms of LSD1 were detected [42] in the pulled down samples. When the samples were probed for VDR, VDR was detected in both the vehicle- and 1,25-D_3_-treated samples (Fig. 2a). This indicates that LSD1 and VDR interact indirectly by belonging to the same multi-protein complex. Furthermore, the presence of VDR in the nucleus of untreated cells is consistent with the ligand-independent repressive role of NRs [46, 47], which can be rescued by 1,25(D)_3_-mediated transactivation. Overall, these results suggest that LSD1 and VDR belong to the same nuclear transcriptional complex, independently from the presence of 1,25-D_3_.

### LSD1 regulates VDR activity in a gene-specific manner

Since LSD1 and VDR belong to the same transcriptional complex, we sought to investigate whether LSD1 can regulate the transcription of VDR target genes. As 1,25-D_3_ affects cell proliferation by regulating CDKN1A expression [12] and E2F target genes [14], we quantified Cdkn1a and E2f1 transcript levels in BC1A cells transfected with siRNA targeting LSD1, or scrambled siRNA (Additional file 1: Figure S3A), and treated with 1,25-D_3_. Furthermore, since 1,25-D_3_ is metabolized by CYP24A1 [48], and S100g is a key player in 1,25-D_3_-mediated calcium absorption [3], both Cyp24a1 and S100g transcript levels were quantified via qRT-PCR. LSD1 knockdown significantly reduces *Cdkn1a* levels and modestly downregulates *E2f1* transcript **(**Fig. 2b). Similarly, 1,25-D_3_ treatment significantly downregulates *Cdkn1a*, with a similar pattern observed for *E2f1*, suggesting that VDR actively represses Cdkn1a and E2f1 transcription at this time point. Interestingly, LSD1 knockdown inhibits 1,25-D_3_-mediated repression on both genes, leading to increased *E2f1* and *Cdkn1a* gene expression (Fig. 2b). A minimal response to 1,25-D_3_ treatment was still detected after 24 h (Additional file 1: Figure S4A), suggesting that E2f1 and Cdkn1a are fast responders to 1,25-D_3_. These data indicate that at basal levels, LSD1 contributes to the maintenance of Cdkn1a and E2f1 transcription, hence the downregulation of both transcripts upon LSD1 knockdown. Conversely, in the presence of 1,25-D_3_, LSD1 mediates a rapid VDR-dependent downregulation of Cdkn1a and E2f1; in the absence of LSD1, this inhibitory effect is lost, therefore relieving Cdkn1a and E2f1 transcriptional inhibition with subsequent increase in the transcript levels.

In contrast, 1,25-D_3_ treatment upregulates *Cyp24a1* and *S100g* transcripts at 24 h, while combination of LSD1 knockdown and 1,25-D_3_ treatment reduces *Cyp24a1* and *S100g* levels of more than 50% (Fig. 2c). These results indicate that in this context, LSD1 acts as a coactivator for VDR, hence the reduction of Cyp24a1 and S100g transcript levels in LSd1 knockdown cells treated with 1,25-D_3_. Furthermore, *Cyp24a1* is not significantly induced at 4 h while *S100g* accumulation is significantly higher in the knockdown-treated cells at 4 h (Additional file 1: Figure S4B), suggesting that Cyp24a1, and to a lesser extent S100g, are late responders to 1,25-D_3_ stimulation.

Overall, LSD1 appears to have a dual regulatory function for VDR, with a degree of time and locus specificity for different VDR target genes.

### LSD1 affects cell viability

**Fig. 3:**
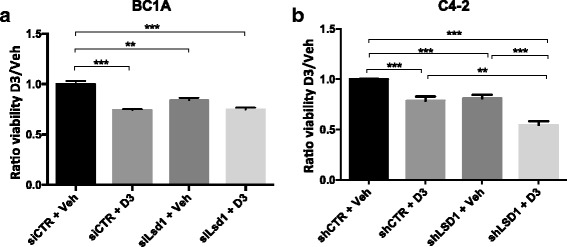
Viability of **a** BC1A and **b** C4-2 cells as measured via cell count upon LSD1 knockdown and 1,25-D_3_ treatment. Each *bar* represents the mean of at least three biological replicates, and the *Y*-axis indicates the percentage of viable cells compared to the control. From *left* to *right*, in both graphs, the *columns* indicate shCTR + Veh, shCTR + 100 nM 1,25-D_3_, shLSD1 + Veh, and shLSD1 + 100 nM 1,25-D_3_. Statistical significance was evaluated with one-way ANOVA and Tukey post hoc correction (***p < 0.001, **p < 0.01, *p < 0.05)

The effect of altered levels of LSD1 on cell viability was tested on BC1A and C4-2 cells. BC1A and C4-2 cells retain minimal 1,25-D_3_ responsiveness, as measured by a reduction in cell viability (Fig. 3). In absence of treatment, LSD1 knockdown leads to a modest but significant reduction in viability in BC1A and C4-2 cells; however, after 72 h of exposure to 100 nM 1,25-D_3_, LSD1 knockdown further enhanced the response to 1,25-D_3_ in C4-2 cells but not in BC1A cells (Fig. 3). These results indicate that LSD1 might play a combinatorial role with VDR in regulating cell viability; however, it appears that other factors contribute to 1,25-D_3_ resistance in BC1A cells.

### LSD1 recruits transcriptional complexes on TSS regions upon VDR activation

In order to evaluate the mechanism of 1,25-D_3_ resistance in BC1A cells, chromatin immunoprecipitation (ChIP) was performed. LSD1 binding was quantified at the Cdkn1a, E2f1, Cyp24a1, and S100g promoters (Additional file 1: Figure S5). LSD1 is detected and significantly enriched over IgG controls at all loci (Additional file 1: Figure S6); however, LSD1 accumulation is not altered by 1,25-D_3_ treatment, suggesting that LSD1 works in concert with, or modulates the activity of, other components of the transcriptional complex to modulate VDR transcriptional effects.

**Fig. 4:**
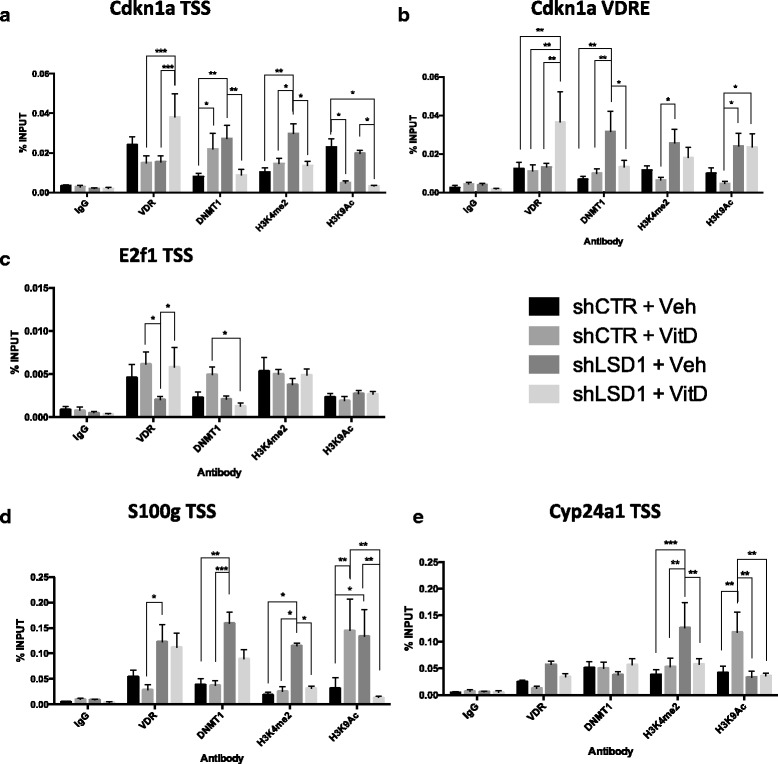
ChIP analysis of BC1A cells stably transfected with shLSD1 lentiviral vector and treated for 24 h with 100 nM 1,25-D_3_. Each *bar* indicates the percentage of binding relative to INPUT and represents the mean of at least three biological replicates with SEM. The *columns* indicate, from *left* to *right*, shCTR + Veh, shCTR + 1,25-D_3_, shLSD1 + Veh, and shLSD1 + 1,25-D_3_. The basal levels of the following protein/histone marks were evaluated, from *left* to *right*, in each graph: *IgG*, *VDR*, *DNMT1*, *H3K4me2*, and *H3K9Ac*. The regions analyzed were **a** Cdkn1a TSS, **b** Cdkn1a VDRE, **c** E2f1 TSS, **d** Cyp24a1 TSS, and **e** S100g TSS. IgG was used as a control for non-specific binding/enrichment. Statistical significance was calculated using one-way ANOVA and Tukey post hoc correction (***p < 0.001, **p < 0.01, *p < 0.05)

To identify the factors involved in VDR-mediated transcriptional program, ChIP analysis was performed using the BC1A cells stably transfected with either scrambled short hairpin control (BC1A-shCTR) or short hairpin targeting *Lsd1* (BC1A-shLSD1) in presence and absence of 1,25-D_3_ (Additional file 1: Figure S3B, C, Fig. 4). We evaluated the binding levels of VDR, as primary mediator of 1,25-D_3_ function, and DNMT1, since LSD1 activity demethylates and stabilizes DNMT1 [33, 49] and since 1,25-D_3_ alters DNA methylation [27] in PCa. Lastly, we evaluated the abundance of the activation marks H3K4me2 and H3K9Ac. Twenty four hour treatment with 100 nM of 1,25-D_3_ increases DNMT1 binding and reduces H3K9Ac at the Cdkn1a TSS (Fig. 4a). Interestingly, LSD1 knockdown also increases DNMT1 binding at both Cdkn1a loci, consistent with reduced *Cdkn1a* levels, but also increases H3K4me2 levels (Fig. 4a, b), consistent with the LSD1 activity as demethylating enzyme for H3K4 [30]. Combination of 1,25-D_3_ treatment and LSD1 knockdown significantly increases VDR and reduces DNMT1 binding (Fig. 4a, b) at both Cdkn1a loci, while increasing H3K9Ac at the Cdkn1a-VDRE locus, suggesting increased transcription as indicated by Cdkn1a mRNA data. Interestingly, 1,25-D_3_ treatment and LSD1 knockdown reduce H3K4me2 and H3K9Ac levels at the Cdkn1a TSS (Fig. 4a). Since VDR can actively recruit other histone lysine demethylases to modulate transcription [50–53], low levels of LSD1 might facilitate this process. Furthermore, VDR was shown to recruit HDACs to the promoter of target genes [54], which seems to occur in an LSD1-independent manner.

Although E2f1 mRNA levels closely mirror *Cdkn1a* pattern, E2f1 TSS binding landscape slightly differs from Cdkn1a TSS. Similar to Cdkn1a, 1,25-D_3_ treatment and LSD1 knockdown increase VDR and reduce DNMT1 binding, consistent with increased gene transcription (Fig. 4c). However, H3K9Ac and H3K4me2 levels are not altered in any condition, suggesting that histone alterations might occur at a different time point or at distal loci surrounding the E2f1 gene.

Overall, these results suggest that in the absence of ligand, LSD1 maintains Cdkn1a expression by preventing DNMT1 binding and demethylating H3K4 at the Cdkn1a promoter. In the presence of 1,25-D_3_, LSD1 prevents VDR from binding the Cdkn1a and E2f1 TSS and stabilizes DNMT1 favoring its binding at the TSS.

1,25-D_3_ stimulation does not significantly change VDR levels at the S100g and Cyp24a1 TSS (Fig. 4d, e) suggesting that although short-time treatments show VDR accumulation at the TSS, VDR might shift to multiple distal VDR-responsive elements (VDREs) [55, 56] after 24 h of exposure to 1,25-D_3_. LSD1 knockdown significantly increases DNMT1 binding at the S100g TSS (Fig. 4e) and H3K4me2 levels at the S100g and Cy24a1 TSS (Fig. 4d, e), as expected from the LSD1 demethylase activity. 1,25-D_3_ treatment increases H3K9Ac levels at the S100g and Cyp24a1 TSS (Fig. 4d, e), consistent with increased transcript levels. Combination of LSD1 knockdown and 1,25-D_3_ treatment reduces H3K9Ac and H3K4me2 levels at the S100g and Cyp24a1 TSS (Fig. 4d, e), supporting qRT-PCR data show reduced *S100g* and *Cyp24a1* transcript levels.

These results suggest that at the S100g TSS, LSD1 limits HDAC activity and leads to increased H3K9Ac, in accordance with the observations made in LSD1 knockout embryos [33]. At the S100 and Cyp24a1 TSS, LSD1 demethylates H3K4 and in cancer cells [30],

These results suggest that LSD1 promotes VDR-mediated upregulation of Cyp24a1 and S100 by maintaining H3K9Ac levels.

### LSD1 affects DNA methylation of VDR-targeted genes

**Fig. 5:**
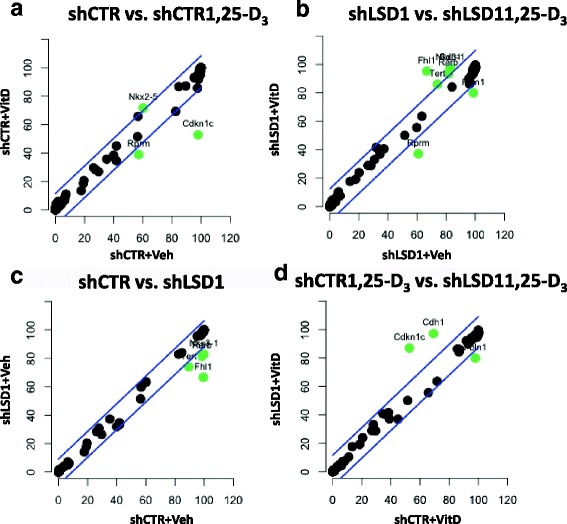
Visual representation of the methylation changes observed using the Qiagen methylation arrays. A linear model was built to identify differentially methylated regions and the 95% confidence intervals calculated and plotted (*blue lines*); *green dots* show the genes whose methylation significantly differs between the selected conditions. Each quadrant reflects the results listed in Additional file 1: Table S1. **a** Contribution of vitamin D at basal conditions. **b** Contribution of LSD1 at basal conditions. **c** Contribution of vitamin D in knockdown conditions (shLSD1). **d** Contribution of LSD1 in the presence of vitamin D

Since LSD1 knockdown reduces Cdkn1a transcript levels with a concomitant increase in DNMT1 binding levels, the methylation status of CpG dinucleotides across the Cdkn1a TSS region was evaluated. BC1A-shCTR and BC1A-shLSD1 cells were treated for 24 h with 100 nM of 1,25-D_3_ prior DNA extraction and bisulfite sequencing. The number of methylated residues across the TSS did not significantly change (chi-square test, *p* = 0.3764) (Additional file 1: Table S1, Additional file 1: Figure S7). Therefore, in order to evaluate whether LSD1 and VDR regulate the methylation status of other cancer-related genes, a PCR array containing methylation-specific probes for 94 genes involved with PCa progression was used. LSD1 knockdown reduces the methylation levels of Fhl1 (− 32.9%), Nkx3.1 (− 17.1%), Rar-beta (− 17%), and Tert (− 15.5%) (Fig. 5c, Additional file 1: Table S2B). These results support LSD1 function in stabilizing DNMT1 by demethylating DNMT1 lysine residue in position 1096 [33]. 1,25-D_3_ treatment reduced the methylation levels of Cdkn1c and Rprm of 45.2 and 18.3%, respectively (Fig. 5a, Additional file 1: Table S2A). At the same time, combination of 1,25-D_3_ treatment and LSD1 knockdown reverses the reduction in methylation caused by 1,25-D_3_ treatment in the Cdkn1c and Chd1 genes, increasing the methylation levels of 34.3 and 28%, respectively, while reducing the methylation of Fbln1 of 18% (Fig. 5d, Additional file 1: Table S2D). Interestingly, 1,25-D_3_ treatment in knockdown cells mostly increased the methylation levels of the genes analyzed (Fig. 5b, Additional file 1: Table S2B), suggesting that VDR might promote secondary post-translational modification that stabilize DNMT1 [57–61] and promote DNA methylation at specific loci. Overall, these results indicate a relationship between LSD1 and DNMT1 in regulating VDR-dependent DNA methylation in PCa.

### LSD1 and DNMT1 status correlate with genome-wide alterations in clinical samples

**Fig. 6:**
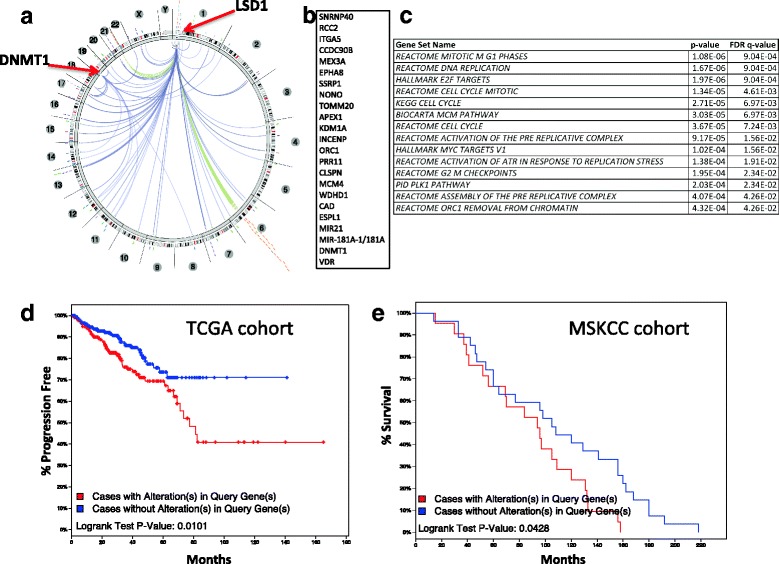
Graphical overview of the alterations in the LSD1/DNMT1/VDR signature. The Regulome Explorer was used to identify genes correlating with LSD1/DNMT1 status, followed by functional enrichment analysis and survival analysis on two independent TCGA datasets. **a** Circos plot showing the genes correlating with LSD1 and DNMT1 status. **b** List of the genes in the LSD1/DNMT1/VDR signature. **c** Functional enrichment analysis of the genes in the LSD1/DNMT1/VDR signature indicating pathway name and origin, *p* value, and FDR-corrected *q* value. **d**, **e** Kaplan-Meier plot indicating progression-free survival in patients with primary tumor (**d**) or overall survival in patients with recurrent metastatic tumor (**e**). The *red lines* indicate patients with altered LSD1/DNMT1/VDR signature (*z* score > ± 2), and the *blue lines* indicate patients whose signature is not altered (*z* score between − 2 and + 2). Statistical significance was calculated via log-rank test with a threshold of *p* < 0.05

Since LSD1, DNMT1, and VDR appear to have interconnected roles in PCa, we sought to identify an LSD1/DNMT1-centered genome-wide signature that has clinical relevance, measured as progression-free survival (PFS) and overall survival (OS). We leveraged data from The Cancer Genome Atlas (TCGA), which include 333 primary PCa samples [62] and 61 metastatic [63]. We first utilized the Regulome Explorer tool to identify genes whose status (expression, methylation, CNV, mutations, protein levels) correlated with LSD1 and DNMT1. Three different statistical approaches were used to evaluate significant correlations based on the data type (details are in the “Methods” section) (Fig. 6a). The top 20 statistically significant genes (Fig. 6b, Additional file 2: File 1), together with VDR, were used to evaluate whether altered expression of this signature correlates with altered patient survival. We queried TCGA survival data for two different patient cohorts, primary and metastatic [63], through the cBioPortal [64]. The results show that patients with altered VDR/LSD1/DNMT1 signature had a shorter PFS in cohort 1 (Fig. 6d) and a shorter OS in cohort 2 (Fig. 6e). To better understand which pathways were affected by our signature, we performed the functional enrichment analysis utilizing Broad’s GSEA pathways. The results indicate that the genes in our signature belong to pathways mainly involved in controlling proliferation and cell survival, including DNA replication, M-G1 phase, cell cycle, activation of pre-replicative complex, G2-M checkpoints, and chromatin remodeling (Fig. 6c). Overall, these data suggest that the alterations in the VDR/LSD1/DNMT1 signature lead to alterations in key cellular pathways, whose function directly affects patients’ outcome.

## Discussion

LSD1 is a key histone demethylase enzyme whose expression directly reflects disease status in numerous tumor types. In the TRAMP model, the expression of LSD1 and VDR is already elevated at early age where most of the initial tumorigenic transformations occur, leading to hyperplasia of the prostate. Furthermore, high levels of LSD1 and VDR proteins promote tumor growth in the CWR22 xenograft model of PCa, indicating a potential combinatorial role of VDR and LSD1 in promoting tumor establishment and progression. We hypothesized that LSD1 plays a key role in the regulation of the VDR-dependent transcription in PCa cells and tested LSD1 as a VDR coregulator. For the first time, LSD1 is identified as dual coregulator, corepressor, and coactivator, for the VDR.

LSD1 is needed for appropriate embryonic development, and LSD1 knockout is embryonically lethal [33, 65]. LSD1 role in cell growth is also observed in cancer cells, and these data were corroborated by our results, where LSD1 knockdown significantly reduced PCa cell viability. Moreover, genomics analyses of the LSD1-VDR-DNMT1 signature suggest that the mechanism by which LSD1 affects cell growth is through transcriptional control of genes involved with cell cycle and proliferation. Interestingly, the combination of 1,25-D_3_ treatment and LSD1 knockdown further increased 1,25-D_3_ antiproliferative effect in C4-2 cells but not in BC1A cells. These results suggest that in BC1A cells, the VDR axis could be inhibited by other coregulatory proteins. Therefore, we sought to investigate the role of LSD1 in regulating the VDR transcriptional complex in BC1A cells. Although we do not show direct physical interaction between LSD1 and VDR, immunoprecipitation experiments show that LSD1 and VDR belong to the same transcriptional complex, suggesting that changes within its components can modulate the direction and magnitude of gene transcription. We utilized the ChIP assay to evaluate the binding of LSD1, VDR, and DNMT1 and the levels of H3K4me2 and H3K9Ac at the TSS of VDR target genes. As LSD1 binds similarly to all loci, increased DNMT1 recruitment is observed in knockdown cells at Cdkn1a and Cyp24a1 TSS. In parallel, LSD1 demethylates lysine 4 on histone H3 [30], supporting the increased H3K4me2 levels observed upon LSD1 knockdown at all but E2f1 promoter. This suggests a locus- and time-specific action of LSD1, which was not captured at the E2f1 TSS. Furthermore, it was previously shown that ligand-activated VDR can recruit HDAC1 and HDAC3 to the promoter of target genes [54]. We indeed observed a reduction in H3K9Ac levels after 1,25-D_3_ treatment at the Cdkn1a TSS and at the TSS of Cyp24a1 and S100g upon LSD1 knockdown and 1,25-D_3_ treatment*.* These results support lower *Cdkn1a* mRNA levels observed after 1,25-D_3_ treatment and in *Cyp24a1* and *S100g* mRNA after 1,25-D_3_ treatment in LSD1 knockdown cells. Overall, these results support the hypothesis that in our system, LSD1 modulates gene transcription by altering the recruitment of the transcriptional machinery and the chromatin conformation in the promoter of VDR target genes.

LSD1 was indeed shown to interact with FOXA1 [66] to regulate androgen receptor (AR)-dependent transcription, and it is known to complex with HDACs, RCOR1 [67], GSE1 [67], RBPJ [68], and ZBP1 [69] in a cell-specific manner, supporting the hypothesis that LSD1 might act as a “regulatory anchor” to form and maintain regulatory complexes on the promoter of VDR target genes. Furthermore, as PCa is hormonally driven, the crosstalk between AR and VDR in prostate cancer was previously investigated, reporting that AR activation inhibits VDR-mediated transcription through the activity of different coregulatory proteins such as prohibitin, ARA70, or ZNF366 [70–72]. As LSD1 has a dual role as corepressor and coactivator for AR and VDR, we believe that the crosstalk between AR/LSD1/VDR is gene- and locus-specific, with potentially different roles in androgen-sensitive vs. castration-recurrent PCa. In this context, ChIP-Seq analysis of LSD1 binding sites upon AR activation and/or 1,25-D_3_ treatment would be useful to profile the potential LSD1-mediated transcriptional feedback between AR and VDR.

A glimpse into the mechanistic function of LSD1 on the chromatin structure comes from studies in breast cancer cells demonstrating that LSD1 is involved in 1,25-D_3_-mediated chromatin looping on the CDKN1A promoter. Knockdown of LSD1 significantly reduced the percentage of looping over untreated cells [73], suggesting that demethylation by LSD1 is essential for correct spatial rearrangement of the transcriptional machinery. Furthermore, LSD1 knockdown in breast cancer cells blocked the estrogen-mediated transcription of *TFF1* and *GREB1* by inhibiting the interaction between the TFF1 and GREB1 loci and the interchromatin granules containing transcription-related factors [74]. Overall, these data suggest that LSD1 regulatory functions are common to numerous transcription factors and that transcriptional activation or repression is a coordinated event, finely regulated by temporal and spatial factors.

In this study, DNMT1 is identified as an important player in the VDR-LSD1 network. LSD1 knockdown causes a drastic reduction in the methylation levels of Fhl1, Nkx3.1, Rarb, and Tert, supporting the stabilizing effect of LSD1 on DNMT1. However, it is interesting to note that upon LSD1 knockdown and 1,25-D_3_ treatment, there is an increase in methylation in Cdh1 and Cdkn1c. This suggests that active VDR might favor secondary post-translational modifications that stabilize DNMT1 [60, 61] and consequently promote DNA methylation. In this regard, ChIP sequencing experiments for the DNMT proteins would be useful to profile their dynamic activity across the genome upon LSD1 knockdown.

Complementary correlative computational approaches were used to investigate the role of LSD1, DNMT1, and VDR in a wider, clinical context. Interestingly, genes that belong to the VDR/LSD1/DNMT1 signature enrich pathways that regulate different stages of cell proliferation, including activation of pre-replicative complexes, cell cycle transition, and transcriptional regulation through E2f. Lastly, the translational significance of these results is demonstrated by the fact that alterations in this signature significantly correlate with shorter survival in primary and metastatic prostate cancer.

## Conclusions

**Fig. 7:**
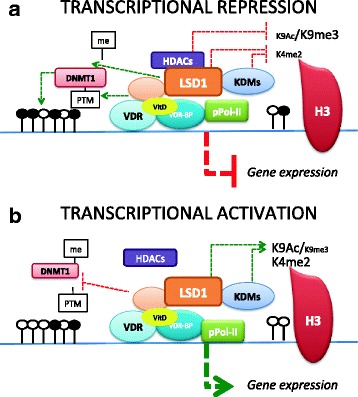
Graphical representation of the model for VDR/LSD1/DNMT1 activity in **a** loci where LSD1 acts as coactivators vs. **b** loci where LSD1 acts as a corepressor. *me* methyl residue, *PTM* post-translational modification, *VDR-BP* VDR binding partner (i.e., RXR), *KDMs* non-LSD1 lysine demethylases, *H3* histone 3, *K9Ac* acetylated lysine position 9, *K9me3* trimethylated lysine position 9, *K4me2* dimethylated lysine position 4, *pPol-II* phosphorylated RNA polymerase II

In conclusion, we hypothesize a model of VDR/LSD1-mediated gene regulation in which LSD1 acts as both coactivator and corepressor for VDR in the presence of 1,25-D_3_. In the presence of active VDR, the LSD1-containing complex represses gene transcription in a locus-specific manner by stabilizing DNMT1, reducing H3K4me2, while HDACs reduce H3K9Ac levels, potentially in favor of the repressive mark H3K9me3. VDR contributes to this mechanism by recruiting secondary epigenetic modifiers to alter the methylomic landscape or histone tail status (Fig. 7a). In loci where LSD1 promotes transcription, target selection changes leading to maintained H3K4me2 levels in the presence of 1,25-D_3_ and LSD1 potentially targeting H3K9me3, favoring H3K9Ac. Furthermore, DNMT1 is demethylated by LSD1, which leads to a less stable protein and consequentially lower methylation of VDR target genes and increased gene transcription (Fig. 7b). Overall, the coordinated action and composition of the regulatory complexes will define the epigenetic status and the transcriptional output, resulting in locus-specific transcriptional activation or repression.

As numerous studies correlate 1,25-D_3_ serum levels with disease status, these results suggest that our LSD1, DNMT1, and VDR signature might mediate an epigenetic lesion that correlates with an altered genomic signature in patient samples. Thus, the contribution of LSD1 in cancer progression could be underestimated and pharmacological inhibition of LSD1 would potentially affect numerous steroid receptor-mediated endocrine pathways; many of which are often altered in cancer.

## Additional files

Additional file 1: Figure S1: mRNA levels of Lsd1 in age-matched WT and TRAMP prostates. Each point is the average of five biological replicates with SEM. (** = *p* < 0.001, *** = *p* < 0.0001). **Figure S2**. Representative IHC images of CWR22 tumors stained for LSD1 (left) or VDR (right). Mice are divided into HIGH (top) or LOW (bottom) depending upon protein abundance. **Figure S3**. Knockdown efficiency. A) siRNA and B, C) shRNA against Lsd1 in BC1A (A, B) and C4-2 cells (C) measured by qRT-PCR-TaqMan and D, F) cropped WB. (***p < 0.001, **p < 0.01, *p < 0.05, Student’s t test). **Figure S4**. qRT-PCR for A) E2f1 and Cdkn1a at 24 h and B) Cyp24a1 and S100g at 4 h. Each bar is the mean of at least three biological replicates with SEM, as fold changes of 1,25-D3 vs. Veh treated samples (***p < 0.001, **p < 0.01, *p < 0.05). **Figure S5**. Map of the regions analyzed via ChIP (in red) for Cdkn1a, E2f1, S100g, and Cyp24a1. **Figure S6**. ChIP analysis for LSD1 in BC1A cells. Each bar is the mean of at least three biological replicates with SEM. X-axis indicates the locus, Y-axis indicates the fold enrichment over INPUT, IgG was used as control for aspecific binding. Statistical significance was calculated comparing LSD1 with IgG within each condition using Student’s t test. (**p < 0.01, *p < 0.05). **Figure S7**. Map of the CpG sites analyzed via bisulfite sequencing at the Cdkn1a TSS. Each shade of gray represents the average methylation level across four biological replicates. The position of each site is indicated at the bottom. **Table S1**. Results from bisulfite sequencing. **Table S2**. LSD1- and vitamin D-driven changes in DNA methylation in PCa-related genes. **Table S3**. Primer sequences used for ChIP analysis. (DOCX 20587 kb)

Additional file 2: Raw results from the regulome explorer analysis for LSD1 and DNMT1. (CSV 36 kb)

## Abbreviations

1,25(OH)_2_-D_3_: Vitamin D
ChIP: Chromatin immunoprecipitation
DNMT1: DNA methyltransferase 1
LSD1/KDM1A: Lysine-specific demethylase 1A
OS: Overall survival
PCa: Prostate cancer
PFS: Progression-free survival
TCGA: The Cancer Genome Atlas
VDR: Vitamin D receptor
