# Supplementary material for: LSD1 dual function in mediating epigenetic corruption of the vitamin D signaling in prostate cancer
Source: Clin Epigenetics. 2017 Aug 11;9:82. doi: 10.1186/s13148-017-0382-y (PMC5553900; doi:10.1186/s13148-017-0382-y)
Supplement: Supplementary file 1 — mRNA levels of Lsd1 in age-matched WT and TRAMP prostates. Each point is the average of five biological replicates with SEM. (** = p < 0.001, *** = p < 0.0001). Figure S2. Representative IHC images of CWR22 tumors stained for LSD1 (left) or VDR (right). Mice are divided into HIGH (top) or LOW (bottom) depending upon protein abundance. Figure S3. Knockdown efficiency. A) siRNA and B, C) shRNA against Lsd1 in BC1A (A, B) and C4-2 cells (C) measured by qRT-PCR-TaqMan and D, F) cropped WB. (***p < 0.001, **p < 0.01, *p < 0.05, Student’s t test). Figure S4. qRT-PCR for A) E2f1 and Cdkn1a at 24 h and B) Cyp24a1 and S100g at 4 h. Each bar is the mean of at least three biological replicates with SEM, as fold changes of 1,25-D3 vs. Veh treated samples (***p < 0.001, **p < 0.01, *p < 0.05). Figure S5. Map of the regions analyzed via ChIP (in red) for Cdkn1a, E2f1, S100g, and Cyp24a1. Figure S6. ChIP analysis for LSD1 in BC1A cells. Each bar is the mean of at least three biological replicates with SEM. X-axis indicates the locus, Y-axis indicates the fold enrichment over INPUT, IgG was used as control for aspecific binding. Statistical significance was calculated comparing LSD1 with IgG within each condition using Student’s t test. (**p < 0.01, *p < 0.05). Figure S7. Map of the CpG sites analyzed via bisulfite sequencing at the Cdkn1a TSS. Each shade of gray represents the average methylation level across four biological replicates. The position of each site is indicated at the bottom. Table S1. Results from bisulfite sequencing. Table S2. LSD1- and vitamin D-driven changes in DNA methylation in PCa-related genes. Table S3. Primer sequences used for ChIP analysis. (DOCX 20587 kb) [file 13148_2017_382_MOESM1_ESM.docx]

LSD1 DUAL FUNCTION IN MEDIATING EPIGENETIC CORRUPTION OF THE VITAMIN D SIGNALING IN PROSTATE CANCER

^1^Sebastiano Battaglia, ^2^Ellen Karasik, ^2^Bryan Gillard, ^2^Jennifer Williams, ^3^Trisha Winchester, ^2^Michael Moser, ^3^Dominc J Smiraglia, ^2^Barbara A. Foster

Roswell Park Cancer Institute, ^1^ Center For Immunotherapy, ^2^ Department of Pharmacology and Therapeutics, ^3^ Department of Cancer Genetics. Elm and Carlton St, 14263, Buffalo, NY, USA.

To whom correspondence should be addressed to: SB Email: [sebastiano.battaglia@roswellpark.org](mailto:sebastiano.battaglia@roswellpark.org) , BF: Email: [barbara.forster@roswellpark.org](mailto:barbara.forster@roswellpark.org)

**SUPPLEMENTARY FIGURES and TABLES:**

**
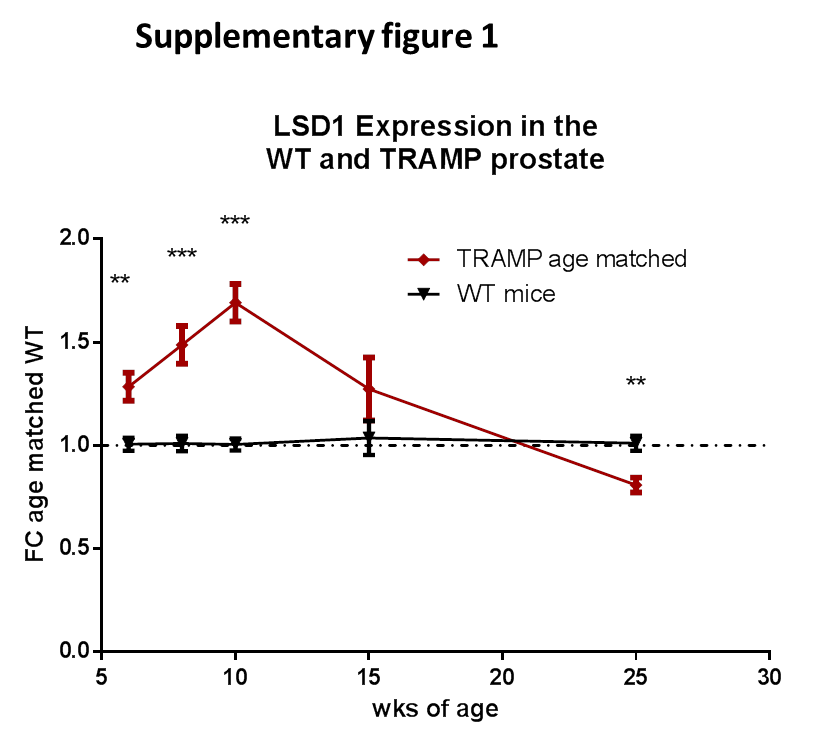
**

**Supplementary Figure 1:** mRNA levels of Lsd1 in age-matched Wild Type (WT) and TRAMP prostate specimens. Red line indicates fold changes in the TRAMP samples compared to the age-matched WT samples. X-axis indicates the age of the mouse. Each data point is the average of 5 biological replicates with SEM. (** = p<0.001, *** = p<0.0001)

**Supplementary Figure 2**

**Supplementary Figure 2:** Representative immunohistochemistry (IHC) images of CWR22 tumors resected from xenograft mice and stained for LSD1 (left) or VDR (right). Mice are divided into two groups depending upon the intensity of the staining: HIGH (top images) or LOW (bottom images).

**Supplementary Figure 3**

**Supplementary Figure 3:** Knockdown efficiency shown at the mRNA and protein levels. **A)** silencing RNA and **B-C)** short hairpin RNA against Lsd1 in BC1A (**A-B**) and C4-2 cells (**C**) measured by qRT-PCR-TaqMan and **D-F)** cropped western blotting. Red bars indicate fold changes of Lsd1 mRNA against the control transfected cells (black bars) and for western blot PNCA was used as endogenous control for the nuclear extract. (***- p<0.001, **- p<0.01, *- p<0.05, Student’s t-Test)

**Supplementary Figure 4**

**Supplementary Figure 4:** qRT-PCR for **A)** E2f1 and Cdkn1a at 24 hours and **B)** Cyp24a1 and S100g at 4 hours. Each bar is the mean of at least three biological replicates with SEM, showing the fold changes of the treated (+1,25-D_3_) vs the vehicle treated (-Veh) samples. Columns indicate, from left to right, siCTR+Veh, siCTR+1,25-D_3_, siLSD1+Veh, siLSD1+1,25-D_3_. (***- p<0.001, **- p<0.01, *- p<0.05)

**Supplementary Figure 5**

**
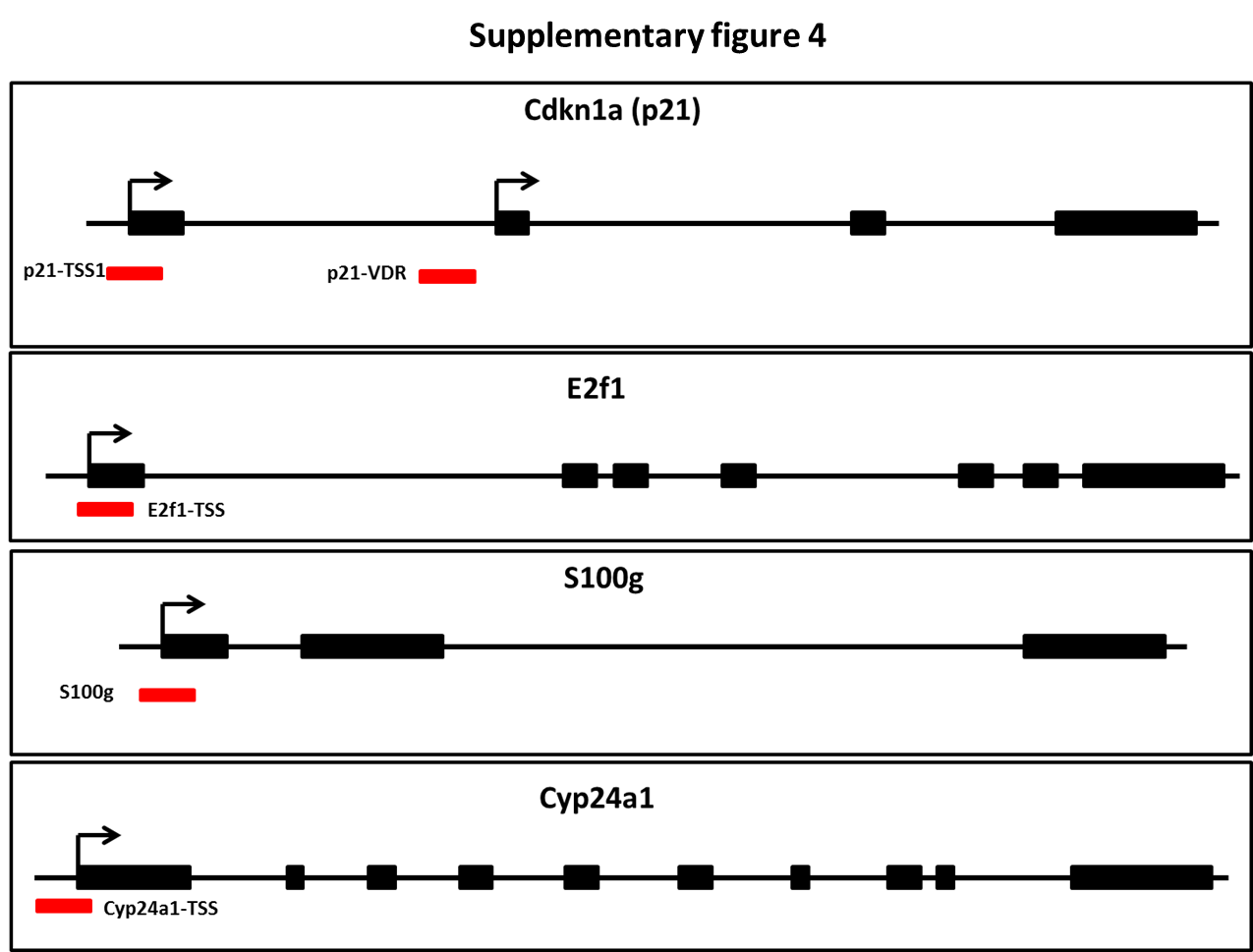
**

**Supplementary Figure 5:** Map of the regions amplified by the primers designed for ChIP analysis (in red) for Cdkn1a, E2f1, S100g and Cyp24a1.

**Supplementary Figure 6**

**
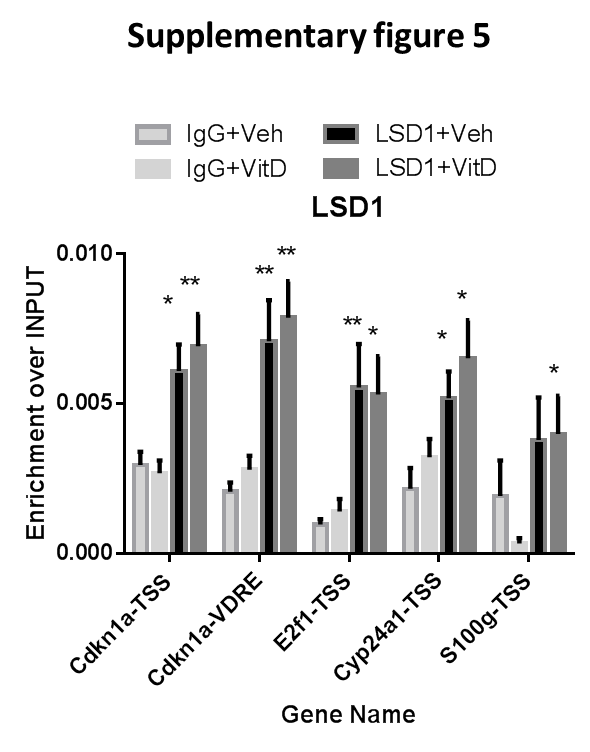
**

**Supplementary Figure 6:** ChIP analysis for LSD1 in BC1A cells treated for 24 hours with 100nM vitamin D. Each bar is the mean of at least three biological replicates with SEM. X axis label indicates the locus analyzed, Y axis indicates the fold enrichment over INPUT, IgG was used as control for aspecific binding. Columns indicate, from left to right, IgG pulldown + Veh, IgG pulldown + 1,25-D_3_, LSD1 pulldown + Veh, LSD1 pulldown + 1,25-D_3_. Statistical significance was calculated comparing LSD1 data with IgG data within each condition using Student’s t.test. (**- p<0.01, *- p<0.05)

**Supplementary Table 1:** Table showing the results from bisulfite sequencing. From left to right, columns indicate cell line condition and treatment, the total number of methylated residues and the total number of non-methylated residues in the region analyzed.

**Supplementary Figure 7:** Map of the CpG sites analyzed via bisulfite sequencing at the Cdkn1a TSS. Each shade of gray represents the average methylation level across 4 biological replicates. The position of each site is indicated at the bottom of the graph with position 1 being the first nucleotide of the primers used. Treatment conditions are on the left side.

**Supplementary Table 2:** ***LSD1 and vitamin D driven changes in DNA methylation in PCa-related genes.*** DNA was digested with methylation sensitive and methylation dependent enzymes, the pattern of digestion detected via qRT-PCR-SYBRGREEN dictates the changes in methylation. Quantitative analysis was done using the SABiosciences analysis template file. Comparisons are indicated in bold on top of each quadrant. Columns indicate, from left to right per each quadrant: percentage of methylation changes in the selected comparison, initial methylation levels and methylation levels after treatment. Only changes larger than +/- 10% are shown. **A)** Contribution of vitamin D at basal conditions, **B)** contribution of LSD1 at basal conditions, **C)** contribution of vitamin D in knockdown conditions (shLSD1), **D)** contribution of LSD1 in the presence of vitamin D.

**Supplementary Table 3:** Primer sequences used for ChIP analysis.
